# PanACoTA: a modular tool for massive microbial comparative genomics

**DOI:** 10.1093/nargab/lqaa106

**Published:** 2021-01-12

**Authors:** Amandine Perrin, Eduardo P C Rocha

**Affiliations:** Microbial Evolutionary Genomics, CNRS, UMR3525, Institut Pasteur, 28, rue Dr Roux, Paris 75015, France; Sorbonne Universite, College doctoral, F-75005 Paris, France; Bioinformatics and Biostatistics Hub, Department of Computational Biology, Institut Pasteur, USR 3756 CNRS, 28, rue Dr Roux, Paris 75015, France; Microbial Evolutionary Genomics, CNRS, UMR3525, Institut Pasteur, 28, rue Dr Roux, Paris 75015, France

## Abstract

The study of the gene repertoires of microbial species, their pangenomes, has become a key part of microbial evolution and functional genomics. Yet, the increasing number of genomes available complicates the establishment of the basic building blocks of comparative genomics. Here, we present PanACoTA (https://github.com/gem-pasteur/PanACoTA), a tool that allows to download all genomes of a species, build a database with those passing quality and redundancy controls, uniformly annotate and then build their pangenome, several variants of core genomes, their alignments and a rapid but accurate phylogenetic tree. While many programs building pangenomes have become available in the last few years, we have focused on a modular method, that tackles all the key steps of the process, from download to phylogenetic inference. While all steps are integrated, they can also be run separately and multiple times to allow rapid and extensive exploration of the parameters of interest. PanACoTA is built in Python3, includes a singularity container and features to facilitate its future development. We believe PanACoTa is an interesting addition to the current set of comparative genomics tools, since it will accelerate and standardize the more routine parts of the work, allowing microbial genomicists to more quickly tackle their specific questions.

## INTRODUCTION

Low cost of sequencing and the availability of hundreds of thousands of genomes have made comparative genomics a basic toolkit of many microbiologists, geneticists, and evolutionary biologists. Many bacterial species of interest have now over 100 genomes publicly available in the GenBank RefSeq reference database, and a few have more than ten thousand. This trend will increase with the ever decreasing costs of sequencing, the availability of long-read technologies, and the use of whole-genome sequencing in the clinic for diagnostics and epidemiology. As a result, researchers that would like to use available assemblies are faced with extremely large amounts of data to analyze. Comparative genomics has spurred important contributions to the understanding of the organization and evolution of bacterial genomes in the last two decades (1,2). It has become a standard tool for epidemiological studies, where the analysis of the genes common to a set of strains — the core or persistent genome — provides unrivalled precision in tracing the expansion of clones of interest (3,4). The use of routine sequencing in the clinic will further require rapid and reliable analysis tools to query thousands, and soon possibly millions of genomes from a single species (5). Population genetics also benefits from this wealth of data because one can now track in detail the origin and fate of mutations or gene acquisitions to understand what they reveal of adaptive or mutational processes (6). Finally, genome-wide association studies have been recently adapted to bacterial genetics, to account for variants in single nucleotide polymorphism and gene repertoires (7). They hold the promise of helping biologists to identify the genetic basis of phenotypes of interest. Given the high genetic linkage in bacterial genomes, these studies may require extremely large datasets to detect small effects. More specifically, reverse vaccinology is also a noteworthy application of these pangenomics methods, to identify novel potential antigens among core surface-exposed proteins of a given clade (8).

The availability of large genomic datasets puts a heavy burden on researchers, especially those that lack extensive training in bioinformatics, because their analysis implicates the use of automatic processes, efficient tools, extensive standardization and quality control. Many tools have been recently developed to make rapid searches for sequence similarity with excellent recall rates for highly similar sequences (9–11).

Other tools provide methods to rapidly cluster large numbers of sequences in families of sequence similarity, to get the families common to a set of genomes, to align them, or to produce their phylogeny, four cornerstones of comparative genomics. A number of recent programs have recently been published that include some of these tools to compute bacterial pangenomes (for a review, see (12)). Many of these programs compute alignments and clusters of families using programs that are very fast. They use tools that make some compromises between accuracy and speed, such as DIAMOND (9), USEARCH (13) and CD-HIT (14). The latter is used, among others, by Roary (15), which is currently the most popular tool to compute pangenomes, and Panaroo (16), a very recent tool aiming at reducing the impact of erroneous automated annotation of prokaryotic genomes. BPGA (17), using USEARCH or CD-HIT to cluster proteins, also provides some downstream analyses. PanX (18), which has an outstanding graphical interface, uses DIAMOND to search for similarities among genes.

More recently, SonicParanoid introduced the use of the highly efficient and accurate program mmseqs2 to build pangenomes, and PPanGGOLiN used the same tool to provide a method to statistically class pangenome families in terms of their frequency (19–21) . Some recent programs also use graph-based approaches to further refine the pangenomes, such as PPanGGOLiN and Panaroo (16). For that matter, the analysis of a dataset of 319 *Klebsiella pneumoniae* genomes by both tools provided similar results (16). Some tools, such as PIRATE (22) have also been recently developed to cluster orthologues between distant genomes. However, all these programs lack some or all of initial and final steps that are essential in comparative genomics, including download, quality control, alignment and phylogenetic inference. This spurred the development of PanACoTA (PANgenome with Annotations, COre identification, Tree and corresponding Alignments). To take advantage of the vast amount of genomic information publicly available, one needs six major blocks of operations. (i) Gather a set of genomes of a clade automatically. This requires some quality control, to avoid drafts with an excessive number of contigs. It is also often convenient to check that the genomes are not too redundant, to minimize computational cost and biases due to pseudo-replication. On the other side, it is important to check that genomes are neither too unrelated, to eliminate genomes that were misclassified in terms of bacterial species (or the taxonomic organization of relevance). (ii) Define *a priori* an uniform nomenclature and annotation, without which the calculation of pangenomes and core genomes becomes unreliable for large datasets. (iii) Produce the pangenome, a matrix with the patterns of presence/absence of each gene family in the set of genomes, using an accurate, simple and fast method. (iv) Use the pangenome to identify sets of core or persistent genes. (v) Produce multiple alignments of the gene families of the core or persistent genomes. (vi) Finally, produce quickly a reasonably accurate phylogeny of the set of core/persistent genes. These four collections of data, pangenome, core genome, alignments and phylogenetic tree, are the basis of most microbial comparative genomics studies. At the end of this process, the researcher can produce more detailed analyses, specific to the questions of interest, which often lead to changes such as including/excluding taxa, changing the thresholds of sequence similarity, increasing alignment accuracy, or rebuilding phylogenies using different methods. Such re-definitions can be achieved more efficiently when pipelines are modular and allow to restart the analyses at several key points in the process.

Considering the current availability of pipelines for microbial comparative genomics, we have built one that is modular, easy to setup, uses state-of-the-art tools and allows simple re-use of intermediate results. The goal was to provide a pipeline that allows to download all genomes from a taxonomic group and make all basic comparative genomics work automatically. The pipeline is entirely built in a single language, Python v3, and uses modern methods to facilitate its future maintenance and to limit unwanted behavior. PanACoTA is freely available under the open source GNU AGPL license. Here, we describe the method and illustrate it with an analysis of two datasets of 225 complete and 3980 complete or draft genomes of *K. pneumoniae*. This species is interesting for our purposes because there are many genomes available and it has a very open pangenome (23). The first dataset describes a situation where sequence quality is usually high, and the second illustrates how the method scales-up to a very large dataset where some sequences and assemblies are of lower quality. The procedure is detailed in the Materials and Methods section, whereas the illustration of its use, and how it changes in relation to key options in the two datasets, is detailed in the Results section.

## MATERIALS AND METHODS

**Figure 1.**
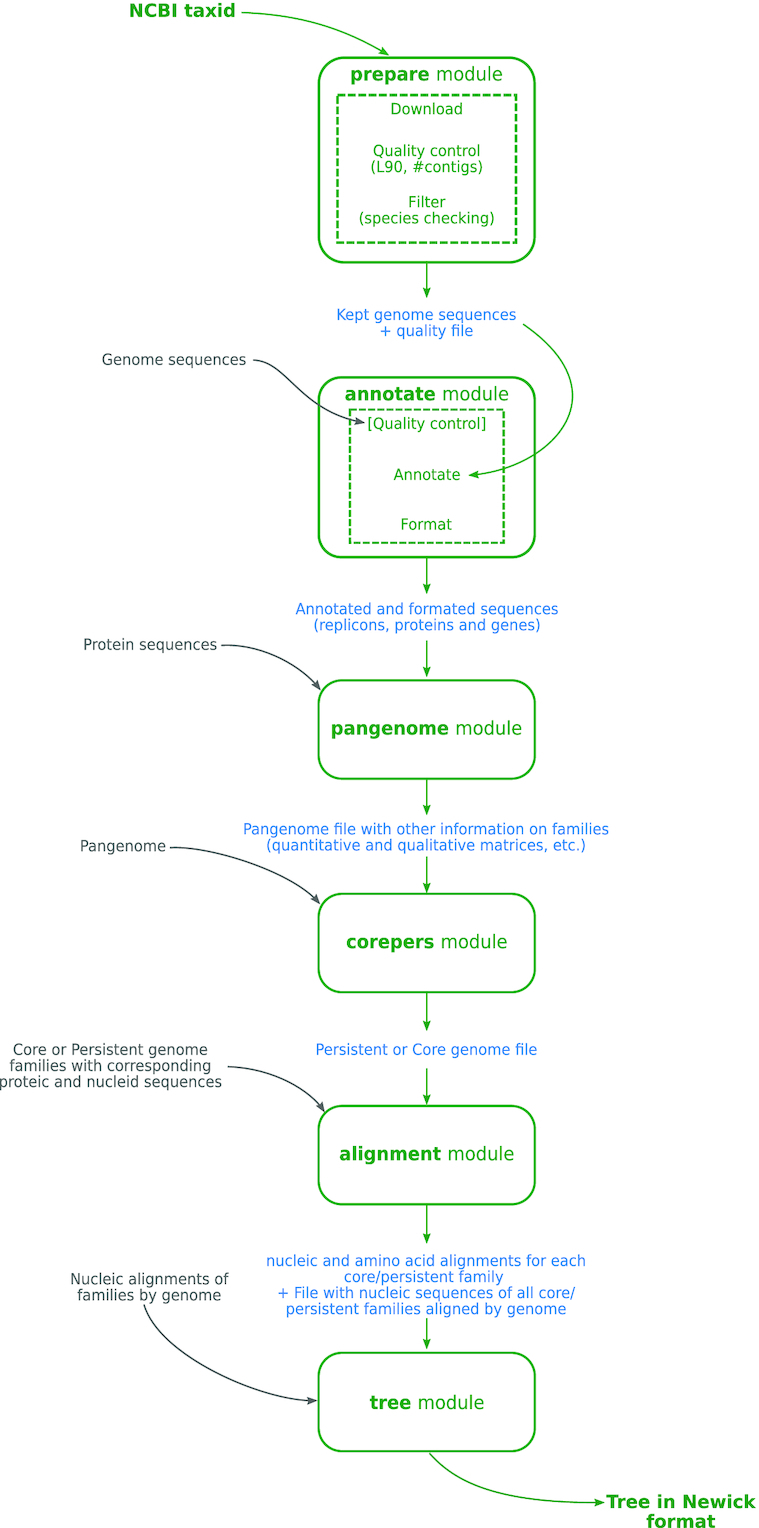
Overview of PanACoTA method.

PanACoTa is implemented in six independent sequential modules, described in the sections below. This allows to start or stop at any step and re-run an analysis with other parameters (see overview in Figure 1 and key parameters in Table 1). It also provides a module *all*, which allows to run all modules in a single-command.

**Table 1.** Key parameters for each module of PanACoTA

Module	Key parameters	Short description	Default values
***prepare***	NCBI species taxid		If user wants to download
	NCBI species		Genomes from NCBI
	- -cutn n	Split contig when there are at least ‘n’ N in a row	5
	- -l90 x	Discard genome(s) with L90 higher than x	100
	- -nbcont x	Discard genome(s) with more than x contigs	999
	- -min_dist x	Discard genome(s) closer than a Mash distance of x	10^-4^
	- -max_dist x	Discard genome(s) with a Mash distance higher than x	0.06
***annotate***	- -l90 x	Discard genome(s) with L90 higher than x	100
	- -nbcont x	Discard genome(s) with more than x contigs	999
	- -prodigal	Use only prodigal instead of Prokka	False
***pangenome***	-i x	Minimum sequence identity to be considered in the same family	0.8
	-c x	Clustering mode (0 for ‘set cover’, 1 for ‘single-linkage’, 2 for ‘CD-Hit’)	1
***corepers***	-t tol	Min % of genomes having at least 1 member in a family to consider the family as persistent	1 (core-genome)
	-M	‘Multiple persistent genome’	False
	-X	‘Mixed persistent genome’	False
***align***	-c file	File containing core genome	
***tree***	-s software	Software to infer phylogeny	IQtree

### Datasets

The first module prepare fetches the compressed non-annotated fasta files assemblies from the NCBI matching a given taxonomy ID using the scripts from ncbi_genome_download library (https://github.com/kblin/ncbi-genome-download).

We use two datasets of *K. pneumoniae* genomes to illustrate how PanACoTA functions. DTS1 contains all complete and draft assemblies from the NCBI refseq database on 10 October 2018. DTS2 is the subset of DTS1 containing only the complete genomes (genomes with assembly_level = Complete Genome, based on the NCBI summary file).

### Quality control procedure

PanACoTA removes assemblies that do not conform with basic requirements in terms of assembly and taxonomy. This is done by the prepare module after downloading the genomes, or by the annotate module before the annotation step (if the user did not use the prepare module).

The first control procedure filters genomes in terms of sequence quality. Since there is usually no standard description of the quality of the sequence assembly in RefSeq genomes, the program infers it from the sequences. First, it is common usage to put stretches of ’N’ to separate contigs in a same fasta sequence. Hence, PanACoTA splits sequences at each stretch of at least a given number of ’N’ to get one fasta entry per contig. Assuming that the user is analyzing genomes from the same species, those genomes should have relatively similar characteristics in terms of number of contigs and length. Hence, PanACoTA calculates the total number of contigs, and the L90 (the minimum number of contigs necessary to get at least 90% of the whole genome). Very high values of these two variables are usually an indication of low quality of sequencing or assembling, resulting in genome exclusion.

The second procedure filters redundant and misclassified genomes. This is done based on the genetic distance between pairs of genomes, as calculated by Mash (24), which can be computed very fast and is accurate for closely related genomes. Mash reduces each genome sequence to a sketch of representative k-mers, using the MinHash technique (25). It then compares those sketches, instead of the full sequences. The Mash distance D strongly correlates with alignment-based measures such as the Average Nucleotide Identity (ANI) based on whole-genome sequence comparisons using the blast algorithm (26): *D* ≈ 1 − *ANI*. For ANI in the range of 90–100%, the correlation with Mash distance is even higher when increasing the sketch size. Since pangenomes are typically computed for a single bacterial species, we are here using Mash to discriminate genomes having at least 94% identity. A few recent programs have been published showing slightly more accuracy than Mash, but we found them too slow for the use as a systematic filter when performing millions of pairwise genome comparisons. For example, using 15 cores, FastANI (27) requires around 1h15 to compare all pairs of 200 genomes (40 000 pairwise comparisons), where Mash with a sketch size of 10^6^ does the task in less than 3 min. The program dRep (28) uses Mash as a pre-filter and then makes more accurate and time-consuming analyses. This is very useful when comparing draft genomes of very different sizes, like metagenomic assembled genomes, but less so for the analysis of within-species complete genomes. Users requiring a finer grade study of ANI may wish to post-analyze their genomes using these programs.

Bacterial species are usually defined as groups of genomes at more than 94% identity (29), which sets the default threshold for D (max_mash_dist = 0.06). On the other extreme, genomes with very high similarity (low Mash distances) provide very similar information. Their exclusion decreases the time required for the analysis and diminishes over-sampling of certain clades. PanACoTA sets min_mash_dist to 10^-4^ by default. This represents one point change every 10 genes, which may be close to the sequencing and assembling accuracy of many draft genomes.

The two procedures, quality control and Mash filtering, are linked together. The information on the number of contigs and L90 is useful to chose the genome that is kept between a pair of very similar genomes. In summary, the control procedure works as follows:

- Genomes with an excessively high number of contigs or L90 are excluded.
- Genomes are primarily sorted by increasing L90 value, and secondarily by increasing number of contigs to produce a list ordered in terms of quality.
- The genomes are compared with Mash. For that, the first genome of the ordered list (the one with best quality) is compared to all the others. The ones which do not obey to the distance thresholds are discarded. The procedure then passes to the subsequent genome in the ordered list (if not rejected before), compares it to all remaining genomes, and discards those not respecting the thresholds. The process continues until the ordered list is exhausted.

The output of the prepare module is a database with the genomes that passed the two steps of the quality control procedure: 3980 genomes for DTS1 and 225 complete genomes for DTS2 (accession numbers in Supplementary Table S1). PanACoTA also provides a file listing the discarded genomes and why they were discarded.

### Annotation

The annotate module provides uniform gene annotation. It takes as input a database of fasta sequences, from the prepare module or provided by the user. If no information is given on the quality control of those genomes (number of contigs and L90), this quality control is done here (see previous section for more information on the quality control step).

**Figure 2.**
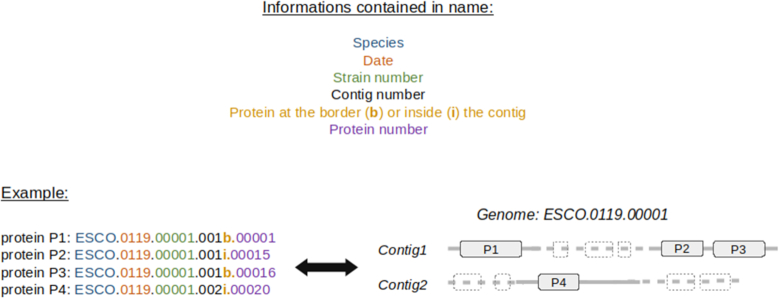
Description of the standard output header format for proteins annotated by PanACoTA.

PanACoTA annotates all genomes with Prokka (30). The latter uses Prodigal (31) to identify gene positions. It then adds functional annotations using a series of programs, including BLAST+ (32) to search for homologs in a database of proteins taken from Uniprot and HMMER3 (33) to search for proteins hitting selected profiles from TIGRFAM (34) and PFAM (35). All annotated sequences are renamed using a standard sequence header format. The header of each gene contains 20 characters and provides human readable information on the genome and contig of the gene, its relative position in the genome and if it is at the border of a contig (see Figure 2).

If the user does not need the functional annotation, the module gives the possibility of running only the gene finding part, i.e. only running Prodigal. For very large datasets it is much faster to use this option and annotate a posteriori only one gene per family of the pangenome using Prokka or more complete annotation systems like InterProScan (36). The output of this step consists in five files per genome: the original sequence, the genes, the proteins (all in fasta format), a gff file containing all annotations and a summary information file.

### Identification of the pangenome

The pangenome module of PanACoTA computes the set of all protein families in the genomes (on the ’Proteins’ folder generated by the annotate module).

The inference of the pangenome involves comparisons between all pairs of proteins, i.e. its complexity is to the square of the number of genes (and thus of genomes). To generate a reliable pangenome in a reasonable time, PanACoTA calls the MMseqs2 suite (20). The mmseqs search module has a very good speed/sensitivity trade-off. In order to reduce time, it uses three consecutive search stages, with increasing sensitivity and decreasing speed. Everything is highly parallelized and optimized on multiple levels. The first step filters up to 99.9% of the sequences by eliminating high dissimilarities, i.e. sequences not having at least two consecutive kmer matches. The second step filters out another 99% of the remaining sequences using an ungapped alignment. This leaves a small amount of sequences to process with an optimized version of the Smith–Waterman alignment, where only scores are calculated, and not the full alignments.

We used the mmseqs cluster module included in MMseqs2 suite, with the default Cascaded clustering option. This module works in two main steps. It first clusters proteins using linclust (37), a linear time protein sequence clustering algorithm as a prefilter. Then, the representative sequences of this first step are handled by the mmseqs search module and clustered. This second step is repeated three times, each time with a higher sensitivity at the mmseqs search algorithm module.

PanACoTA uses the Connected component mode for clustering, because it has provided results consistent with our previous methods. This mode uses transitive connections to merge pairs of homologous genes. Alternatively, two other clustering modes (Greedy Set cover, or Greedy incremental) are available in the pangenome module. Importantly, the tuning of the options of mmseqs2 allows the sequence similarity analyses to be exceedingly fast or extremely sensitive (20). In PanACoTA the user can change the key parameters –min-seq-id and –cluster-mode, and re-run the mmseqs cluster module to explore their effect on the results. More specific mmseqs2 parameters have, for the time being, to be used with the standalone version of the program.

This step outputs files containing one line per family of the pangenome and indicating the gene identifiers, the presence of the gene family (binary matrix), or the number of elements. The latter can be used as input for TreeWAS (38).

Panacota does not take into account synteny between genes in the genomes, which has limited interest in draft genomes. Several programs can do such analyses, e.g. panOCT (39,40), SynerClust (41) or PANINI (42).

### Identification of core and persistent genomes

**Figure 3.**
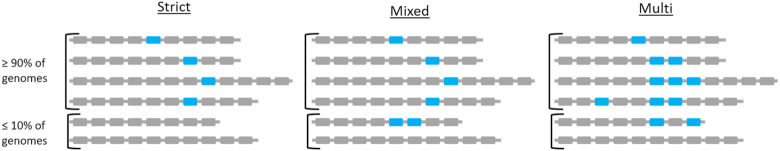
Different types of persistent genomes proposed by PanACoTA, with a threashold of *N* = 90%.

The classification of gene families present in a large number of taxa is done by the corepers module using a file generated by the pangenome module. In early studies, the pangenome matrix was used to identify the gene families present in all genomes in a single copy: the core genome. However, the increase of the number of genomes in the dataset tends to decrease drastically the size of the core genome. This is because sequencing or annotation errors as well as rare deleterious polymorphism in the populations lead to the rapid decrease of the number of core genes with the increase in the number of input genomes. To overcome this problem, one commonly identifies the persistent genome, which is more robust to rare (true or artifactual) variants. PanACoTA defines three types of persistent genomes (see Figure 3):

- Strict-persistent: a family that contains exactly one member in at least N% genomes (*N* = 100 means it is a core-family). This definition is particularly practical to reconstruct phylogenies without having to handle the existence of multiple copies per genome.
- Mixed-persistent: a family where at least N% of the genomes have exactly one member, and other genomes have either zero, either several members in the family. This definition is intermediate between the other two, i.e. it includes the strict-persistent and is included by the multi-persistent.
- Multi-persistent: a family with at least one member in N% of the genomes. This definition is interesting to analyze patterns of diversification of nearly ubiquitous protein families.

The module corepers uses the pangenome instead of a reference genome (whose choice can be questionable). Re-running the module is very fast, because it only requires the re-analysis of the pangenome matrix and can be done multiple times with different parameters.

The output of this module is a file containing the persistent families of proteins.

If the user wants to identify the persistent genome using a statistical approach rather than using fixed thresholds, the gff file generated by annotate module is compatible with PPanGGOLiN (21). This software generates the multi-persistent version of the persistent genome (multigenic families are allowed).

### Multiple alignments of the persistent gene families

The alignment of the persistent gene families is done by the align module using the persistent genome coming from the corepers module, or independently provided by the user. When using the strict-persistent genome, all genes are aligned. When using the other definitions of persistent genomes, some genomes can lack a gene or have it in multiple copies and must be handled before phylogenetic inference. When a genome lacks a member or has more than one member (mixed or multi persistent) of a given gene family, PanACoTA adds a stretch of gaps (‘-’) of the same length as the other aligned genes. Adding a few ‘-’ has little impact on phylogeny reconstruction. For example, it has been showed that adding up to 60% of missing data in the alignment matrix could still result in informative alignments (43). In our experience, when this approach is applied to within-species persistent genomes, it usually incorporates <1% of gaps. The effect of missing data should thus be negligible relative to the advantage of using the phylogenetic signal from many more genes (i.e. in contrast to using the strict-persistent genome). Alignments are more accurate when done at the level of the protein sequence. This has the additional advantage of producing codon-based nucleotide alignments that can be used to study selection pressure on coding sequences. Hence, PanACoTA translates sequences, aligns the corresponding proteins and then back-translates them to DNA to get a nucleotide alignment. This last step constitutes in the replacement of each amino acid by the original codon. Hence, at the end of the process, the aligned sequences are identical to the original sequences.

PanACoTA does multiple sequence alignment using MAFFT (10) as it is often benchmarked as one of the most accurate multiple alignment programs available and one of the fastest (44). It has options that allow to make much faster alignments, at the cost of some accuracy, to handle very large datasets. This loss of accuracy is usually low for very similar sequences as it is the case of orthologous gene families within species, and means that PanACoTA can very rapidly align the persistent genome.

This module returns several output files: the concatenate of the alignments of all families to be used for tree inference, and, for each core/persistent genome family, a file with its gene and protein sequences aligned.

### Tree reconstruction

The phylogenetic inference is done with the tree module of PanACoTA. It uses as input the alignments of the align module or any other alignments in Fasta format.

This is the part that takes most time in the entire pipeline, because the time required for phylogenetic inference grows very fast with the size of the dataset. Even efficient implementations of the maximum likelihood analyses scale with the product of the number of sites and the number of taxa, which is a problem in the case of large datasets (thousands of taxa, with more than ten thousands sites for each one). PanACoTA proposes several different methods to obtain a phylogeny: IQ-TREE (45), FastTreeME (46), fastME (47) and Quicktree (48). According to its needs, the user can choose one of these methods to infer its phylogenetic tree. These trees can be used to build more rigorous phylogenetic inference using methods that are more demanding in computational resources, e.g. by changing the options of IQ-TREE. Whatever the software used, the tree module takes as input a nucleotide alignment in Fasta format (like, for example, the output of align module), and returns at least a tree in Newick format. According to the software and options used, other output files may be generated, like bootstrap trees for example. IQ-TREE also returns the BIONJ tree from which it started tree search, as well as the pairwise distance matrix corresponding to the output tree. Recombination is known to affect phylogenetic reconstruction (49,50). To tackle this problem, some researchers detect and then remove recombination tracts from genomes before inferring the phylogeny. This can be done outside PanACoTA by modifying the multiple alignments before proceeding to the phylogenetic inference. We have not implemented in PanACoTA the detection or exclusion of recombination tracts. Several studies have shown that removing the identifiable recombination tracts tends to distort phylogenetic inference at a larger extent than simply using all the information in the multiple alignments (51,52). This is probably because available methods miss many events of homologous recombination, leading to biases in phylogenetic inference. When relevant, one can use methods that simultaneously infer recombination and phylogenetic history, altough these tend to be computationally costly.

### Implementation and availability

PanACoTA was developed in Python3, trying to follow the best practices for scientific software development (53,54). For that, the software is versioned using git, allowing the tracking of all changes in source code during PanACoTA’s development. It is freely distributed under the open-source AGPL v3 licence (making it usable by many organizations) and can be downloaded from https://github.com/gem-pasteur/PanACoTA. The software can be installed directly from the git repository, or using pip or conda package-management systems. A singularity image, including all needed dependencies, is also hosted via Docker Hub. By downloading this image, the user can run PanACoTA without installing anything. This is of particular use for running on clusters, where there is usually no root access.

Hosting PanACoTA on GitHub allows for issue tracking, i.e. users can report bugs, make suggestions or, for developers, participate to the software improvement. To provide a maintainable and reliable software, we set up continuous integration process: each time a modification is pushed, there is an automatic software installation checking, unit tests are done, and, if necessary, an updated version of the documentation is generated, as well as an update of the docker image on Docker Hub (which can be used as a singularity image as described previously).

As introduced just before, we also provide a complete documentation, including a step by step tutorial, based on provided genome examples, so that the user can quickly get started. It also contains more detailed sections on each module, aiming at helping users to tune all parameters, in order to adapt the run to more specific needs. This documentation also includes a ’developer’ section, addressed to developers wanting to participate in the project.

During its execution, PanACoTA provides logging information, so that user can see real-time execution progress (a quiet parameter is also proposed for users needing empty stdout and stderr). This also provides log file(s) to keep track on what was ran (command-line used, time stamp, parameters used etc.).

## RESULTS AND DISCUSSION

All execution times mentioned in this section correspond to wall clock time on eight CPUs (except when the number of CPUs is given). A summary of all execution times can be found in Table 2.

**Table 2.** Summary of execution times by (sub)module

MODULE	STEP	DTS1 (3980 genomes)	DTS2 (225 genomes)
**prepare**	*Downloading*	1 h (5805 genomes)	3 min (266 genomes)
	*Quality control*	<4 min	∼15 s
	*Filter*	20 min	∼1 min
**annotate**	*With Prokka*	5 days	10 h
	*With Prodigal*	6 h	30 min
**pangenome**		30 min	1 min
**corepers (1 CPU)**		1 min	5 s
**align**	*Strict persistent*	3 h	10 min
	*Mixed-persistent*	7 h	11 min
**tree (IQ-TREE2) (28 CPUs)**	*Strict-persistent*	7 h (40 GB RAM)	3 min 10
	*Mixed-persistent*	24 h (90 GB RAM)	3 min 30

### Download and preparation of genome sequences

**Figure 4.**
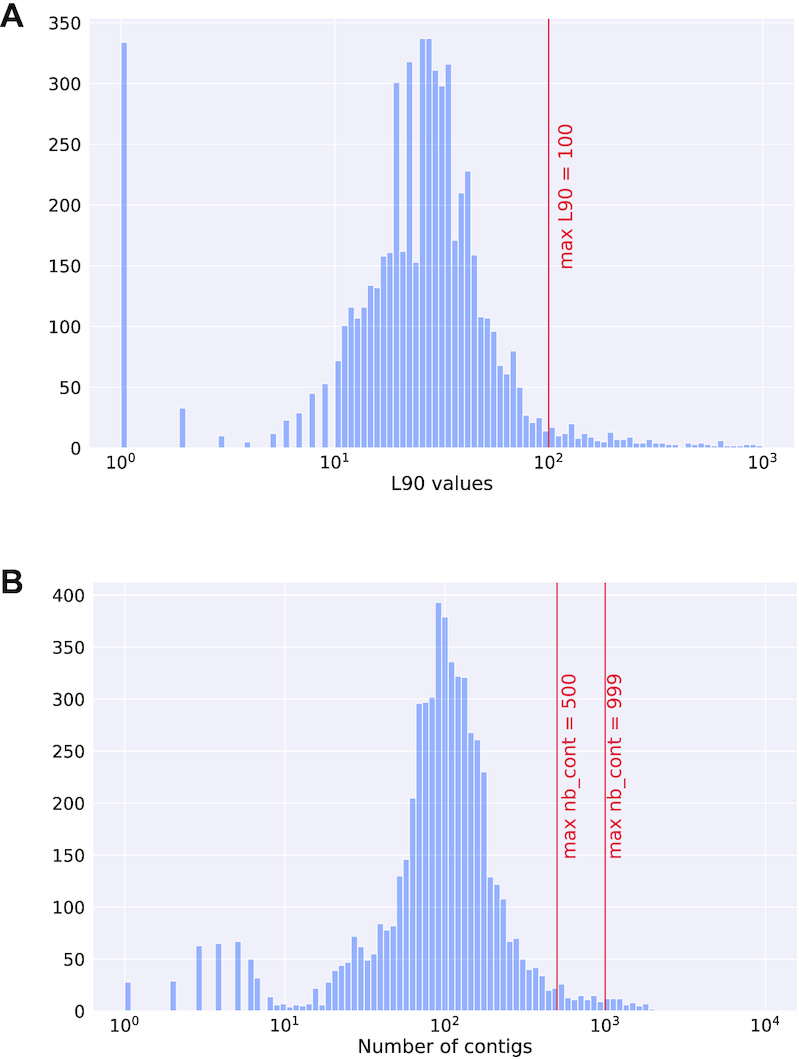
Histograms describing the features of the 5805 *Klebsiella pneumoniae* genomes downloaded from Refseq. (**A**) Distribution of L90 values. (**B**) Distribution of the number of contigs per genome.

The first module of PanACoTA was used to download all genomes of *K. pneumoniae* using the TaxID 573. It took ∼1 h to download the 5805 *K. pneumoniae* genome sequences (including 266 complete genomes). We used the module annotate to make the quality control (L90 < 100 and number of contigs < 999), which took less than 4 min. This step discarded 233 draft genomes, leaving 5572 for further analysis (see Figure 4). When the threshold on the number of contigs was decreased by half (number of contigs < 500), only 52 more genomes were removed (see Figure 4B). To define the best thresholds to the analysis, the user can preview its dataset quality with a ’dry-run’ of the annotate module. Then, the user can launch the real analysis, from prepare or annotate with the adapted thresholds.

**Figure 5.**
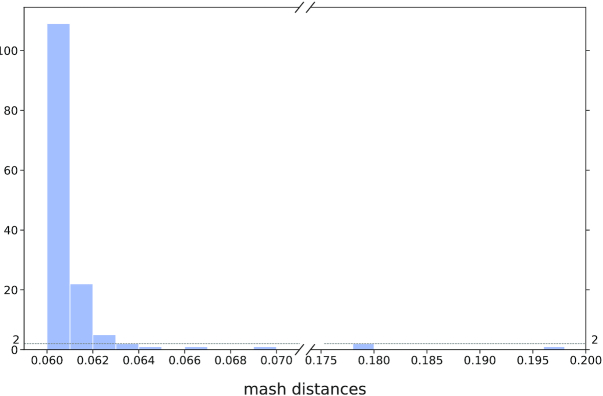
Distribution of Mash distances for the 5572 genomes respecting the L90 and number of contigs thresholds, but having a Mash distance higher than the threshold (0.06).

We removed the very distantly related and redundant genomes using Mash (K-mer size of 21 (default), and sketches of at most 10 000 non-redundant min-hashed *k*-mers). A total of 1592 genomes (including 41 complete genomes) did not respect the distance thresholds (max_mash_dist = 0.06 and min_mash_dist = 1e^-4^). Most (1448) were too similar to other genomes, whereas 144 were too distantly related with the *K. pneumoniae* genomes (Figure 5).

Expert analysis can lead to the definition of narrower ANI values. For example, Kleborate (https://github.com/katholt/Kleborate) (55) defines strong *K. pneumoniae* matches for distances ≤ 0.01 and weak matches between 0.01 and 0.03. In our dataset, Kleborate would have only removed 22 additional genomes, that it identifies as *K. quasipneumoniae subspecies similipneumoniae*. The default method of Panacota, which is designed for any species, is thus consistent with Kleborate results regarding the specific case of *K. pneumoniae* genomes when starting from the NCBI taxonomy ID.

Three genomes showed an ANI <84% identity, meaning they may not even be from the same genus, which emphasizes the necessity of this kind of analysis before computing a pangenome. They were removed from the analysis (GCF_900451665.1, GCF_900493335.1 and GCF_900493505.1). A neighbor-joining tree generated from Mash distance matrix with scikit-bio (https://github.com/biocore/scikit-bio) confirmed the gap between those three genomes and the others (see Supplementary Figure S1, where genomes kept in DTS1 are in green, while those discarded are in red).

**Figure 6.**
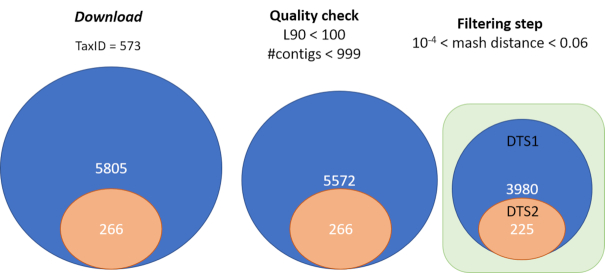
Summary of the procedure to construct DTS1 and DTS2.

Finally, these filters left 3980 genomes in the analysis, with an average of 5307 genes per genome, which will be called the reference database DTS1. Among them, there are 225 complete genomes that form the dataset DTS2 (see Figure 6).

We then proceeded to the functional annotation, which is by far the slowest of the first tasks. The annotation of the genomes with Prokka 1.11 took ∼1 min 50 s per genome, i.e. around 5 days for the whole dataset. For comparison, the annotation using only prodigal 2.60 took less than 6 h (annotation + formatting of all 3980 genomes), i.e. 6 s per genome. Assuming that genes from the same pangenome family have similar functions, one can annotate one protein per family at the end of the process and save considerable time.

### Building pangenomes

The 3980 DTS1 genomes contain 20 765 062 proteins. It took less than 30 minutes to create the protein database in the MMseqs2 format (Release 11-e1a1c), cluster them (with at least 80% identity and 80% coverage of query and target), and retrieve the pangenome matrices. The DTS1 pangenome has 86607 families. Among them, 35 348 (40%) are singletons (found in a single genome), which is concordant with values observed in *Escherichia coli* (56). The pangenome of DTS2, 1 190 485 proteins, was computed in <1 min. It contains 24 473 families, including 8975 (37%) singletons.

**Figure 7.**
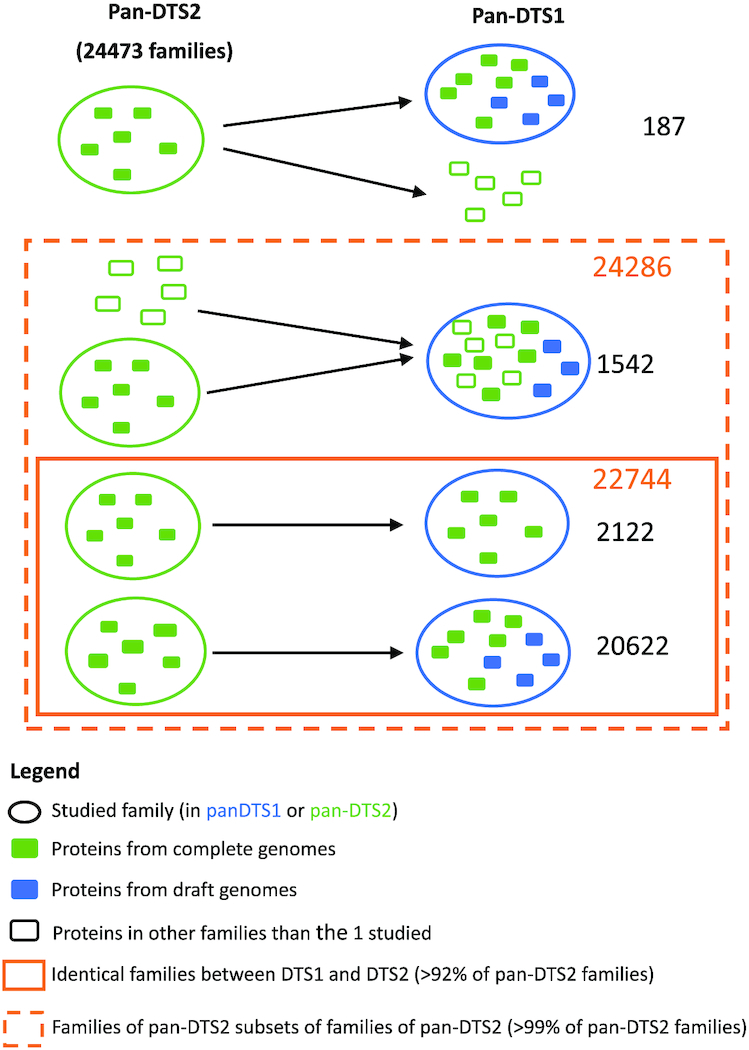
Comparison of the pangenomes generated by PanACoTA for both DTS1 and DTS2.

The comparison of these two pangenomes is interesting because it reveals the robustness of the method to changes in sampling size, as summarized in Figure 7. A total of 2147 families contain only members present in both DTS1 and DTS2. Among these, 2122 families are exactly the same in both pangenomes, whereas only 25 were split in the DTS1 pangenome family relative to the DTS2 pangenome. In most of the latter, they are split in two different families of DTS1. This shows that the clustering procedure is quite robust to the addition of a very large number of genomes.

Most important, 22 744 families (that is more than 92% of all DTS2 families) are identical in DTS1 and DTS2 pangenomes. Identical here means that the DTS2 pangenome gene family is included in a DTS1 pangenome gene family, and the other members of this DTS1 pangenome family are only members of genomes not present in DTS2. Furthermore, around half of the remaining families from the DTS2 pangenome are included in a DTS1 pangenome gene family, which contains a few other proteins from DTS2 genomes. Finally, only 187 gene families of the DTS2 pangenome were split into two or three different families of DTS1 pangenome. In other words, 24 286 families (more than 99%) of DTS2 pangenome are subsets of DTS1 gene families. In conclusion, the construction of pangenome families is robust to large variations in the number of input genomes (see Figure 7).

### Core and persistent genomes

**Figure 8.**
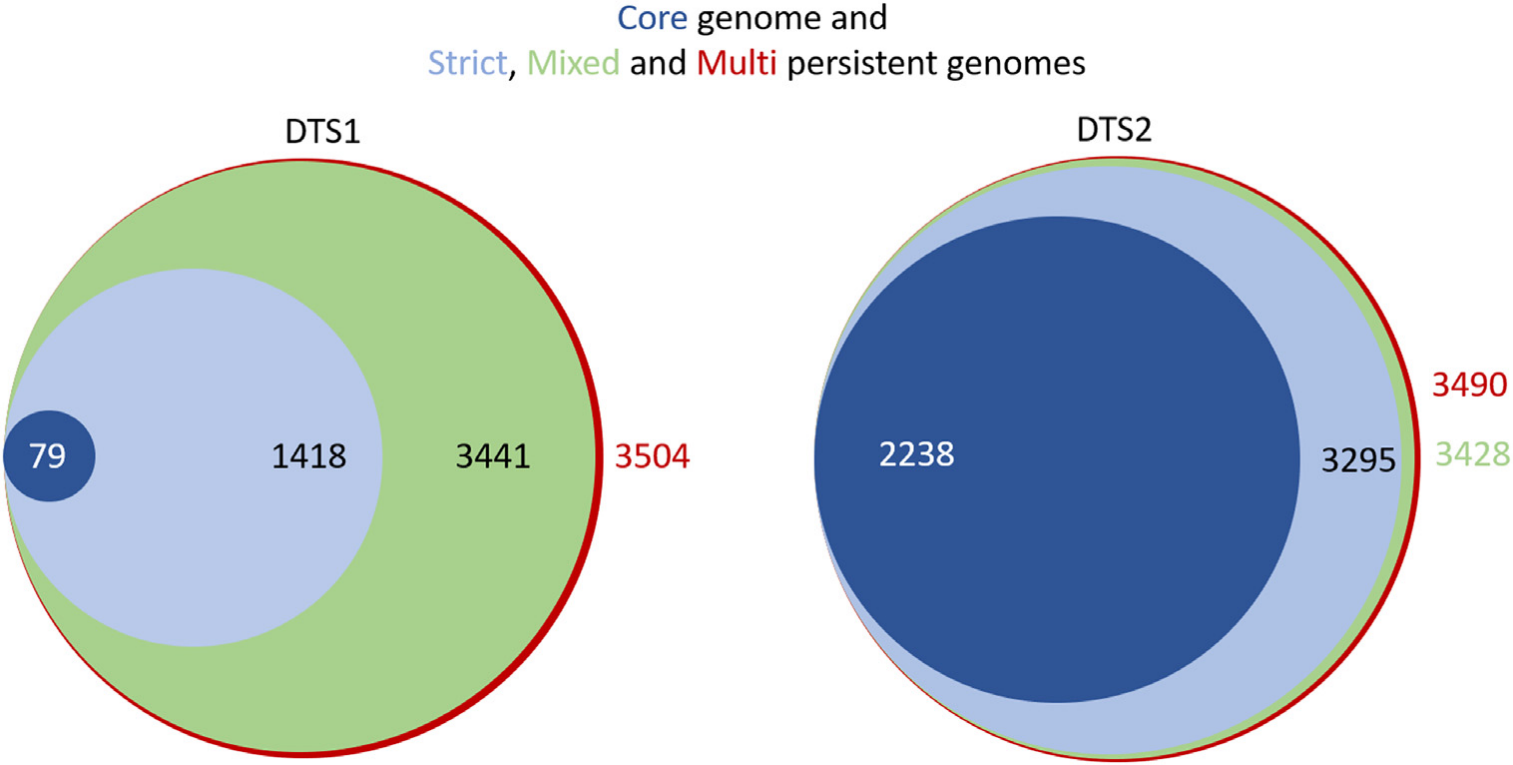
Comparison of the sizes of the core genome and the 3 different types of persistent genomes, for both DTS1 and DTS2. Areas of circles are proportional to the size of the dataset.

This part of the analysis is very fast. Using only one CPU, it took around 1 min to generate a core or persistent genome from DTS1 pangenome. PanACoTA provides a core genome and three different measures of persistent genome (see Figure 3). The strict-persistent genome corresponds to cases when the family is present in a single copy in 99% genomes and absent from the others. In DTS2, the set of complete genomes, the difference between the core and strict-persistent genome is appreciable (2238 versus 3295 families), i.e. the persistent genome is 50% larger (see Figure 8). The difference becomes huge when the analysis is done on the much larger (and less accurate) DTS1 dataset, where the two datasets vary by more than one order of magnitude (79 versus 1418 families). In such large datasets of draft genomes the core genome is not biologically meaningful.

The mixed-persistent genome includes the families present in a single copy in 99% genomes and present (potentially in several copies) or absent from the others. It includes the strict-persistent genome. Its size is close to the latter in the small DTS2 dataset, but much larger in DTS1 (see Figure 8). While the mixed-persistent genome is 65% percent of the average genome in DTS1, the strict-persistent is only 27% percent in the same dataset. This shows the relevance of using definitions of the core genome adapted to the dataset in order to build robust phylogenetic trees or to analyze patterns of genetic diversification and natural selection.

Finally, PanACoTA also computes a multi-persistent genome that includes all gene families present in at least 99% of the genomes, independently of their copy number (see Figure 8). Its analysis reveals many genes encoding regulators, transporters and enzymes that are nearly ubiquitous, but often present in multiple copies. As a rule, this definition is interesting to study gene families present in most genomes, but present in very different copy number. On the other hand, it is typically not very useful for phylogenetic inference.

### Phylogenetic tree inference

PanACoTA ran mafft v.7.467 using --auto option to align all families. For DTS1, it selected the FFT-NS-2 method, while for DTS2, it selected FFT-NS-i method. This was done with both the strict-persistent (1418 families, 3 h) and the mixed-persistent (3441 families, 7h).

PanACoTA used the multiple alignments as input to IQ-TREE multicore version 2.0.6, with the -fast option. For the tree based on the alignment of the strict-persistent (1 438 179 positions), it took around 7 h on 28 CPUs and required 38 GB of RAM. For the tree based on the alignment of the mixed-persistent (3 393 006 positions), it took 24 h using 28 CPUs and required 88 GB of RAM.

We wished to understand the differences in phylogenetic inference in terms of the method used to define the persistent genome (strict and mixed persistent). We computed the patristic distance matrix for each tree and a Pearson correlation test showed that they are strongly correlated (cor = 0.99138, *P* < 2.2*e*^−16^). This shows that the distances provided by the two methods are very similar. Hence, if the strict persistent is large enough to generate a phylogenetic tree, it provides adequate distances between genomes. Aligning all mixed persistent families would just take much more time, for a similar result. However, if one is interested in having a robust tree topology, one should use the larger (and computationally costlier) dataset. Indeed, the analyses of Robinson–Foulds distance with R phangorn package shows a branch-weighted distance of 0.43 and an absolute distance of 2892 (57). This is because some lineages of *K. pneumoniae* account for a large fraction of the data and these parts of the tree require long informative multiple alignments to produce accurate topologies. Accordingly, the differences in topology between the trees using the DTS2 dataset, which have much larger average branch lengths, show much smaller values of topological distances between the two datasets of persistent genome (RF = 78, wRF = 0.027).

## CONCLUSION

PanACoTA is a pipeline for those wanting to test hypotheses or explore genomic patterns using large scale comparative genomics. We hope that it will be particularly useful for those wishing to use a rapid, accurate and standardized procedure to obtain the basic building blocks of typical analyses of genetic variation at the species level. We built the pipeline having modularity in mind, so that users can produce multiple variants of the analyses at each stage. We also paid particularly care with the portability and evolvability of the software. These two characteristics, modularity and evolvability, will facilitate the implementation of novel procedures in the future.

## DATA AVAILIBILITY

The two datasets of *K. pneumoniae* genomes used to illustrate PanACoTA were downloaded from the NCBI refseq. Their accession numbers are indicated in Supplementary Table S1. PanACoTA source code is freely available from https://github.com/gem-pasteur/PanACoTA under AGPLv3 license. More information in the last part of Materials and Methods section.

## Supplementary Material

lqaa106_Supplemental_Files
