# Supplementary material for: PanACoTA: a modular tool for massive microbial comparative genomics
Source: NAR Genom Bioinform. 2021 Jan 12;3(1):lqaa106. doi: 10.1093/nargab/lqaa106 (PMC7803007; doi:10.1093/nargab/lqaa106)
Supplement: lqaa106_Supplemental_Files [file lqaa106_supplemental_files.zip › supplementary figure and table captions.docx]

**supplementary figure: S1-NJ_tree-mash_dist.png**

Neighbor-joining tree generated from Mash distance matrix. Genomes kept in DTS1 are in green, while those discarded are in red.

**supplementary table: table-S1.tsv**

Accession numbers of all genomes (downloaded from the NCBI refseq) used to illustrate PanACoTA in the paper.
