# Supplementary figures and images for: PanACoTA: a modular tool for massive microbial comparative genomics
Source: NAR Genom Bioinform. 2021 Jan 12;3(1):lqaa106. doi: 10.1093/nargab/lqaa106 (PMC7803007; doi:10.1093/nargab/lqaa106)

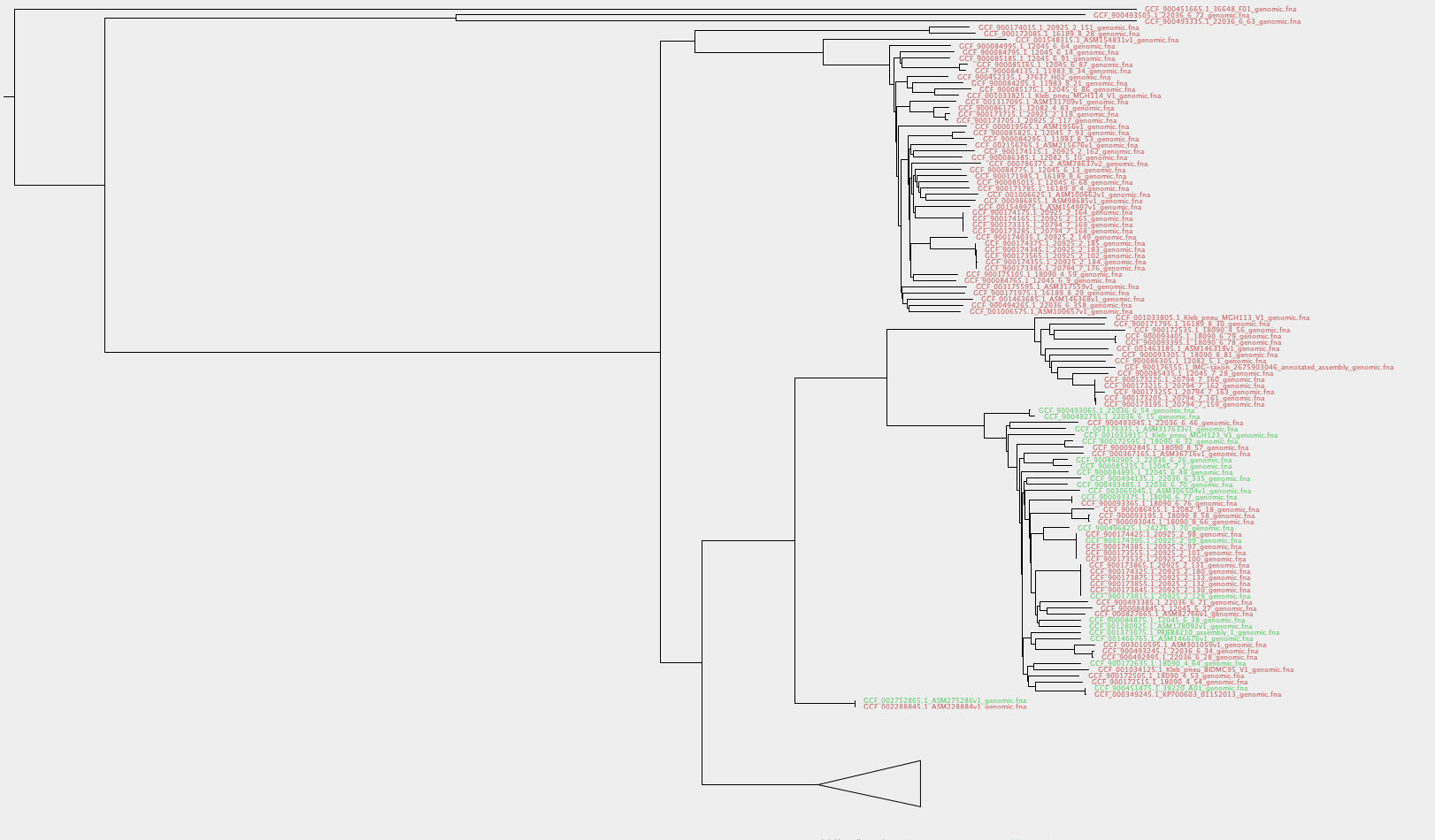

Supplement: lqaa106_Supplemental_Files [file lqaa106_supplemental_files.zip › S1-NJ_tree-mash_dist.png]
